# Association of Scottish Medical Diplomates

**Published:** 1907-03-16

**Authors:** 


### ASSOCIATION OF SCOTTISH MEDICAL DIPLOMATES.

The members and friends of this Association dined together on March 1st at the Trocadero Restaurant, under the chairmanship of the President, Dr. Claude St. Aubyn Farrer. After the usual loyal toasts, "The Association" was proposed by Dr. J. G. Fitzgerald, formerly M.P. for Longford, who referred to the large numbers of those holding diplomas from the Scottish Colleges, and to the need that they, like their English and Irish brethren, should have an organisation by which mutual goodwill and happiness would be promoted. He looked upon the Association as a highly proper and necessary development, and wished it abundant success. In reply, the President gave an account of the progress of the Association and of the work it had accomplished. The authorities were always ready to lend an attentive ear to any representations that might be forwarded to them on behalf of the general body of the diplomates. In so large a constituency there must be many common interests, and probably also some grievances, and it was the purpose of the Association to attend to these and to promote improvements in the professional position of the diplomates. What was wanted was an increase of membership, so that strong and united action might be taken.

